# School Buildings

**Published:** 1879-04

**Authors:** 


### The Bistoury.

ELMIRA, N. Y., APRIL, 1879.

The BISTOURY is published Quarterly, upon the 1st of April, July, October and January, at Fifty Cents a year in advance.

### SCHOOL BUILDINGS.

A radical change is needed in our school-house architecture. Too many children are suffering from impaired vision and health by reason of our faulty school buildings and furniture.

The chief defect consists in that, the light is insufficient and improperly admitted. The buildings are usually two or more stories high, the light being admitted from windows in the side walls. Where the rooms are subdivided into smaller apartments for the primary classes, the light comes from but one side of the room, being too bright for the scholars next the windows, and too dull for those at the opposite side of the apartment. To those who sit facing the light or where it falls upon their side faces, a dazzle is produced when looking up, that requires several moments to remove when the eyes are reapplied to their books. The deficient illumination at the remote side of the room, necessitates the holding of the book nearer the face. To read at this short distance, requires special effort, not only upon the muscles of accommodation, but also increases the labor of the muscles that move the eye. The continual pulling of the internal straight muscles soon elongates the eyeball, producing nearsightedness and often a sort of paralysis of these muscles that gives rise to various optical defects.

School furniture should be carefully adapted to the size of the pupil. Sufficient thought and care is not given to this matter. Usually, the chair or bench is too high, compelling the pupil to bend over his book when committing his lesson. This position favors the flow of blood to the head, causing congestion and consequent painfulness of the eyes.

These imperfections might not be worth the mentioning, were the pupil confined to them but an hour or two daily, but when it is remembered that the larger portion of the day is spent in school, in which the eyes are constantly employed, and the further circumstance that defective vision is becoming alarmingly prevalent among our children, some remedy is called for.

Parents and teachers pay too little attention to the eyes of children. Perhaps this grows out of the fact that the public generally, know comparatively nothing of the existence of the defects mentioned. To call attention to them is the object of this article. An examination of school children's eyes in this and every other city, will reveal that at least five percentum of them are defective, requiring spectacles or skillful treatment to restore the lost function. It is safe to say that most, if not all of such diseases or defects can be immediately traced to our illy constructed school buildings.

What is the remedy, then ? All school buildings, of whatever name or nature, should be but one story high. No side windows should be employed. The light should be admitted from above, through a glass dome. The walls should not be white, because a reflection of light occurs from their surfaces, and reflected light is always unpleasant if not painful to the eye. The walls should be colored therefore, some of the tints of gray or "stone-color" being most agreeable. Ventilation is admirably conducted through this dome, and by means of cold air registers in the floor, while the apartment is rendered cool in summer, warm in winter, and completely prevents annoyance or interruption by what is transpiring outside.

Our schoolhouses are expensive and wretchedly constructed edifices for the purposes intended. The one story plan, with sky-lights or domes, will not only lessen the cost, but will be a means of arresting and preventing the many defects of vision to which our children are now subject, and in many ways improve the sanitary condition of such structures.

Our Board of Education is about to build a primary school building. Will they not take into consideration the many advantages that are offered in a building such as we describe ?

